# Função Endotelial e Hipertensão Arterial

**DOI:** 10.36660/abc.20250179

**Published:** 2025-05-15

**Authors:** Rui Póvoa

**Affiliations:** 1 Universidade Federal de São Paulo São Paulo SP Brasil Universidade Federal de São Paulo, São Paulo, SP – Brasil

**Keywords:** Endotélio, Vasodilatação, Hipertensão

Há alguns anos acreditava-se que o endotélio era simplesmente o revestimento interno dos vasos representando uma simples barreira mecânica. Hoje são profundas as evidências da importância desta fina camada de células na regulação do tônus vascular, e em diversas outras funções, principalmente no crescimento celular, interação entre leucócitos, inflamação tecidual e síntese de substâncias vaso reguladoras.^1^ A regulação do tônus vascular envolve a via de sinalização do óxido nítrico (NO) com fortes propriedades vasodilatadoras, anti-inflamatórias e antioxidantes.^2^

Um endotélio saudável é importante para uma boa homeostase vascular, visto que a disfunção endotelial desempenha um papel significativo na patogênese de muitas doenças tais como a hipertensão arterial e pulmonar, cardiomiopatias, vasculites e de forma bastante objetiva na formação da aterosclerose.^3 , 4^ A biodisponibilidade do NO é considerada como um dos fatores críticos no paciente hipertenso, inclusive em fases iniciais da doença e não só no evento final da disfunção que é a aterosclerose.

A disfunção endotelial está diretamente correlacionada com os eventos cardiovasculares, e a detecção e quantificação se tornam necessárias. Atualmente, a recuperação da integridade vascular se tornou também um novo alvo terapêutico no vasto complexo do grupo de fatores de risco da aterosclerose. Assim, uma estratégia para a avaliação da função endotelial pode fornecer maiores detalhes na prevenção de eventos cardiovasculares. Vários métodos não invasivos foram desenvolvidos, entre os quais a vasodilatação mediada por fluxo (DMF) da artéria braquial, que é a prática mais utilizada na clínica podendo avaliar alterações desde as fases iniciais.^5 , 6^

Neste trabalho elegante de Tessier et al.^7^ foi avaliada a função endotelial utilizando a DMF em pacientes hipertensos e se compararam diversos parâmetros laboratoriais nos grupos de hipertensos resistentes e não resistentes.^7^ Encontraram correlação significativa e positiva entre DMF com o LDL-colesterol e os triglicerídeos. Este tipo de ideia é bastante interessante, pois analisando uma mesma doença, ou fator de risco bem pontual associado, podemos evidenciar outros parâmetros agravantes do risco cardiovascular. Além do mais a avaliação da DMF da artéria braquial tem boa correlação com a função endotelial das artérias coronárias e quando comprometida se associa com a incidência de eventos coronarianos em longo prazo.^8^

Isto foi estudado por Matsuzawa et al. em uma metanálise onde os diversos testes de função endotelial não invasivos, inclusive a DMF previam significantemente eventos cardiovasculares, e todos com magnitude prognóstica semelhante.^9^

Alguns estudos avaliaram a disfunção endotelial na hipertensão arterial, porém os mecanismos envolvidos são bastante complexos devido à associação de doenças também consideradas inflamatórias como a diabetes e as dislipidemias. Em todos estes processos patológicos a relação com o estresse oxidativo é intensa, ocorrendo redução do NO.^10^

Neste estudo de Tessier et al.^7^ as alterações lipídicas, com o encontro da relação do LDL-colesterol e triglicérides com a disfunção endotelial reforça o conceito de que perturbações decorrentes destes estímulos se devem à diminuição da produção de NO e proporções desequilibradas de substâncias vasodilatadoras/vasoconstritoras endoteliais.^7^ Esses desvios são precursores de aumento do risco cardiovascular e sinalizam o início da formação da placa gordurosa. Diversos estudos já mostraram de forma bem evidente que esta lipoproteína de baixa densidade e os triglicérides são fatores de risco bem estabelecidos.^11 - 13^

As alterações lipídicas induzem a disfunção endotelial ao intensificar a inflamação e o estresse oxidativo, que é o passo inicial para a aterosclerose, e podem resultar nas complicações cardiovasculares já bem estabelecidas como o infarto do miocárdio e o acidente vascular cerebral.^14^

No paciente hipertenso seja resistente ou não, a associação destes outros fatores que agridem o endotélio podem ser o início ou acelerar um processo aterosclerótico inicial e evoluir para as grandes complicações vasculares. Apesar de não ser simples as interrelações destes componentes lipídicos e hipertensivos, é importante interromper este processo com o tratamento adequado das diversas comorbidades, atingindo as metas preconizadas nas diretrizes. Seguramente a via final comum da intervenção é a melhora da função endotelial.

Infelizmente, a hipertensão arterial continua sendo uma crise de saúde global, ainda configurando o principal fator de risco modificável no desenvolvimento e progressão das doenças cardiovasculares. Apesar de existirem diversas classes de anti-hipertensivos eficientes e seguros a maioria dos hipertensos não atingem a meta pressórica desejável. Estratégias destinadas ao diagnóstico e prevenção da disfunção endotelial e da redução da produção de NO com certeza terão um impacto positivo na saúde na maioria dos hipertensos.
