# Supplementary material for: Função Endotelial e Hipertensão Arterial
Source: Arq Bras Cardiol. 2025 May 15;122(5):e20250179. [Article in Portuguese] doi: 10.36660/abc.20250179 (PMC12108126; doi:10.36660/abc.20250179)
Supplement: Translation

# Translation: Endothelial Function and Arterial Hypertension

**DOI:** 10.36660/abc.20250179i

**Authors:** Rui Póvoa

**Affiliations:** 1 Brazil Universidade Federal de São Paulo, São Paulo, SP – Brazil

**Keywords:** Endothelium, Vasodilation, Hypertension

A few years ago, it was believed that the endothelium was simply the inner lining of blood vessels, representing a simple mechanical barrier. Today, there is profound evidence of the importance of this thin layer of cells in the regulation of vascular tone and in several other functions, mainly in cell growth, interaction between leukocytes, tissue inflammation, and synthesis of vasoregulatory substances.^1^The regulation of vascular tone involves the nitric oxide (NO) signaling pathway, which has strong vasodilatory, anti-inflammatory, and antioxidant properties.^2^

A healthy endothelium is important for good vascular homeostasis since endothelial dysfunction plays a significant role in the pathogenesis of many diseases, such as arterial and pulmonary hypertension, cardiomyopathies, vasculitis, and, quite objectively, in the formation of atherosclerosis.^3 , 4^The bioavailability of NO is considered one of the critical factors in hypertensive patients, including in the early stages of the disease and not only in the final event of the dysfunction, which is atherosclerosis.

Endothelial dysfunction is directly correlated with cardiovascular events, and detection and quantification are necessary. Currently, the recovery of vascular integrity has also become a new therapeutic target in the vast complex of atherosclerosis risk factors. Thus, a strategy for the evaluation of endothelial function can provide greater details in the prevention of cardiovascular events. Several noninvasive methods have been developed, among which flow-mediated vasodilation (FMD) of the brachial artery, which is the most widely used practice in the clinic and can evaluate changes from the initial phases.^5 , 6^

In this elegant work of Tessier et al.^7^endothelial function was assessed using FMD in hypertensive patients, and several laboratory parameters were compared in the groups of resistant and non-resistant hypertensive patients.^7^They found a significant and positive correlation between FMD and LDL-cholesterol and triglycerides. This type of idea is quite interesting because by analyzing the same disease or a very specific associated risk factor, we can highlight other parameters that aggravate cardiovascular risk. Furthermore, the assessment of FMD of the brachial artery has a good correlation with the endothelial function of the coronary arteries and, when compromised, is associated with the incidence of coronary events in the long term.^8^

This was studied by Matsuzawa et al. in a meta-analysis where several noninvasive endothelial function tests, including FMD, significantly predicted cardiovascular events and all with similar prognostic magnitude.^9^

Some studies have evaluated endothelial dysfunction in arterial hypertension, but the mechanisms involved are quite complex due to the association of diseases also considered inflammatory, such as diabetes and dyslipidemia. In all these pathological processes, the relationship with oxidative stress is intense, with a reduction in NO.^10^

This study of Tessier et al.^7^lipid alterations, with the finding of the relationship between LDL cholesterol and triglycerides and endothelial dysfunction, reinforces the concept that disturbances resulting from these stimuli are due to decreased NO production and unbalanced proportions of endothelial vasodilator/vasoconstrictor substances.^7^These deviations are precursors to increased cardiovascular risk and signal the beginning of the formation of fatty plaque. Several studies have already clearly shown that low-density lipoprotein and triglycerides are well-established risk factors.^11 - 13^

Lipid alterations induce endothelial dysfunction by intensifying inflammation and oxidative stress, which is the initial step towards atherosclerosis and can result in well-established cardiovascular complications such as myocardial infarction and stroke.^14^

In hypertensive patients, whether resistant or not, the combination of these other factors that attack the endothelium can be the beginning or accelerate an initial atherosclerotic process and evolve into major vascular complications. Although the interrelationships between these lipid and hypertensive components are not simple, it is important to interrupt this process with the appropriate treatment of the various comorbidities, achieving the goals recommended in the guidelines. Surely, the final common route of intervention is the improvement of endothelial function.

Unfortunately, hypertension remains a global health crisis and is still the main modifiable risk factor for the development and progression of cardiovascular disease. Although there are several classes of effective and safe antihypertensive drugs, most hypertensive patients do not achieve the desired blood pressure target. Strategies aimed at diagnosing and preventing endothelial dysfunction and reducing NO production will certainly have a positive impact on the health of most hypertensive patients.
